# The transcription factor FOXN3 inhibits cell proliferation by downregulating E2F5 expression in hepatocellular carcinoma cells

**DOI:** 10.18632/oncotarget.9780

**Published:** 2016-06-02

**Authors:** Ji Sun, Hong Li, Qi Huo, Meiling Cui, Chao Ge, Fangyu Zhao, Hua Tian, Taoyang Chen, Ming Yao, Jinjun Li

**Affiliations:** ^1^ Shanghai Medical College, Fudan University, Shanghai, China; ^2^ State Key Laboratrory of Oncogenes and Related Genes, Shanghai Cancer Institute, Renji Hospital, Shanghai Jiaotong University School of Medicine, Shanghai, China; ^3^ Qi Dong Liver Cancer Institute, Qi Dong, Jiangsu Province, China

**Keywords:** FoxN3/CHES1, E2F5, hepatocellular carcinoma

## Abstract

Hepatocellular carcinoma (HCC) is the second leading cause of cancer-related death worldwide, and the mechanisms underlying the development of HCC remain to be elucidated. Forkhead box N3 (FOXN3) is an important member of the FOX family of transcription factors that plays an essential role in several cancers but has not been investigated in HCC. In this study, we demonstrate that FOXN3 is downregulated in human primary HCC tissues compared with their matched adjacent liver tissues. Functional tests of FOXN3 demonstrated that FOXN3 inhibits the proliferation of HCC cells *in vitro* and *in vivo*. Additionally, FOXN3 repressed the mRNA and protein expression of E2F5, a reported potential oncogene, by inhibiting the promoter activity of E2F5. Collectively, our findings indicate that FOXN3 functions as a tumor suppressor in HCC by downregulating the expression of E2F5.

## INTRODUCTION

As the second leading cause of cancer-related death worldwide, hepatocellular carcinoma (HCC) is the worst complication of long-standing chronic liver disease (CLD). The effectiveness of clinical therapies is limited because HCC produces symptoms only at advanced stages [1]. According to the World Cancer Report 2014, there were approximately 782,500 incident cases of liver cancer in 2012. Due to the poor prognosis and limited possibilities for medical intervention, 745,500 deaths occurred worldwide in 2012, of which approximately 50% occurred in China. Most (70% to 90%) primary liver cancers occurring worldwide are HCC [2]. Elucidating the pathogenesis and molecular biology of HCC is crucial for the development of effective therapeutic options [3]. More than 100 putative driver genes are associated with multiple recurrently altered pathways in HCC and may be potential targets for regulation by therapies [4].

Forkhead box N3 (FOXN3), also known as checkpoint suppressor 1 (CHES1), is a member of the forkhead box family, a new family of transcription factors officially named in 2000. The members of the forkhead box family have a common DNA-binding domain and have been associated with organ differentiation, development, cell growth and cancer. The forkhead box family has more than 100 members, which are classified into 15 subclasses (FOXA through FOXS) [5]. FOXN3 belongs to the FOXN subfamily, which has 6 members. FOXN3 has two variants that encode 490 aa and 468 aa human forkhead proteins of undefined function in HCC. Pati *et al.* initially discovered and characterized the protein by screening checkpoint-mutant *Saccharomyces cerevisiae* transfected with human cDNAs to rescue the phenotype [6]. In the present study, we demonstrate that FOXN3 expression is significantly downregulated in several cancer tissues compared with adjacent non-cancerous tissues, including oral squamous cell carcinoma [7] and laryngeal carcinoma [8]. This evidence implies that FOXN3 plays a role in cell proliferation, apoptosis and pathogenesis in human cancer by regulating other genes as a transcription factor. It is important to identify the function and mechanism of FOXN3 to understand the role of FOXN3 in HCC tumorigenesis.

In the present study, we identified E2F5 as a direct target gene of FOXN3. E2F belongs to a family of transcription factors and was named for its function; E2F regulates transcription by binding to a common sequence (TTTSSCGC: S = C or G) known as an E2F site [9]. E2F5 is a member of the E2F family and regulates the expression of genes involved in cell cycle control by directly binding to the promoters of these genes [10]. The present study implies that E2F5 as other E2Fs plays a vital role in apoptosis, senescence, proliferation, the DNA-damage response and DNA repair [11]. Jiang Y determined that E2F5 is commonly upregulated in HCC and that E2F5 knockdown significantly inhibits the growth of HCC cells [10]. However, the upstream mechanism that regulates E2F5 in HCC remains poorly understood.

In the present work, we explore the expression profiles of FOXN3 in HCC and describe a biochemical and genetic interaction between FOXN3 and E2F5, which was originally identified based on its ability to act as a tumor suppressor in HCC.

## RESULTS

### The forkhead transcription factor FOXN3 is downregulated in HCC tissues and its expression is associated with good prognosis in HCC patients

**Figure 1:**
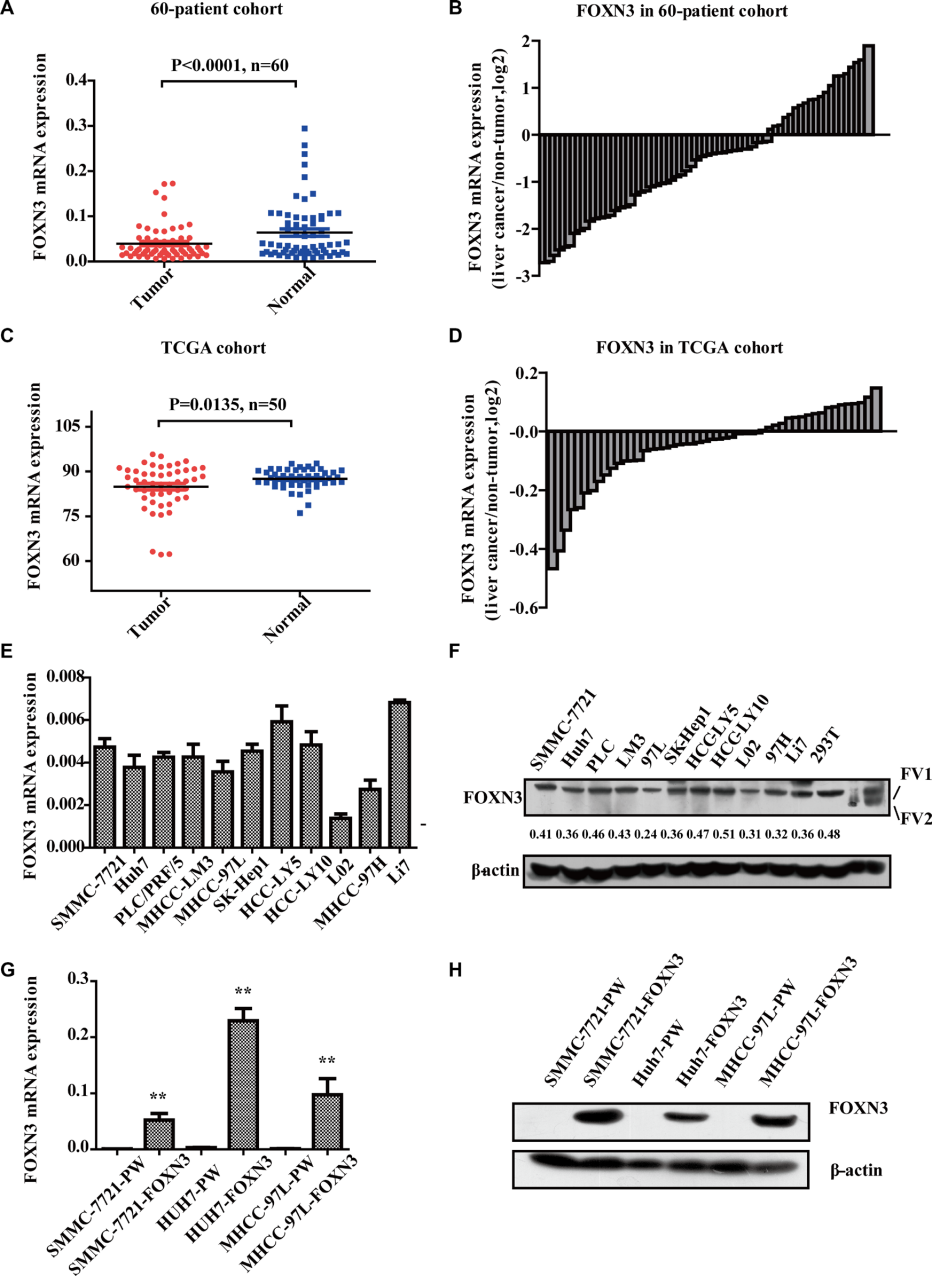
FOXN3 is often downregulated in HCC (**A**) qRT-PCR was performed to detect the expression levels of FOXN3 in the 60-patient cohort. (**B**) The fold change in FOXN3 levels in paired tumorous/non-tumorous tissues in the 60-patient cohort. (**C**) Expression levels of FOXN3 in the TCGA cohort. (**D**) The fold change in FOXN3 levels in paired tumorous/non-tumorous tissues in the TCGA cohort. (**E**) qPCR analysis of FOXN3 expression in HCC cell lines. (**F**) Western blot analysis of FOXN3 expression in HCC cell lines. The protein expression was measured semi-quantitatively with ImageJ software (http://rsb.info.nih.gov/ij/index.html). Relative protein levels were determined by densitometry and calculated as the ratio of the interest protein to its loading control. (**G**) qPCR analysis of FOXN3 expression in HCC cells stably transfected with FOXN3 or control plasmids. (H) Western blot analysis of the FOXN3 protein in HCC cells stably transfected with FOXN3 or control plasmids. *P < 0.05, **P < 0.01.

To investigate the general role of FOXN3 in HCC, we determined the expression of FOXN3 in 60 pairs of HCC tissues and matched non-tumorous liver tissues by quantitative RT-PCR (qRT-PCR). FOXN3 was downregulated in human primary HCC tissues compared with the non-tumorous liver tissues (*P* < 0.0001, Figure 1A and 1B), consistent with analysis from The Cancer Genome Atlas (TCGA) (*P* = 0.0135, Figure 1C and 1D). In addition, overall survival analysis of TCGA data indicated that high FOXN3 expression was closely associated with good prognosis in HCC patients when patients who lived less than five months or longer than seven years were excluded (*n* = 273, log-rank test *P* = 0.046, Supplementary Figure S1A). Consistent with studies of FOXN3 in other cancers, these results imply that FOXN3 plays a key role in the development of HCC.

### FOXN3 inhibits HCC cell proliferation *in vitro*

**Figure 2:**
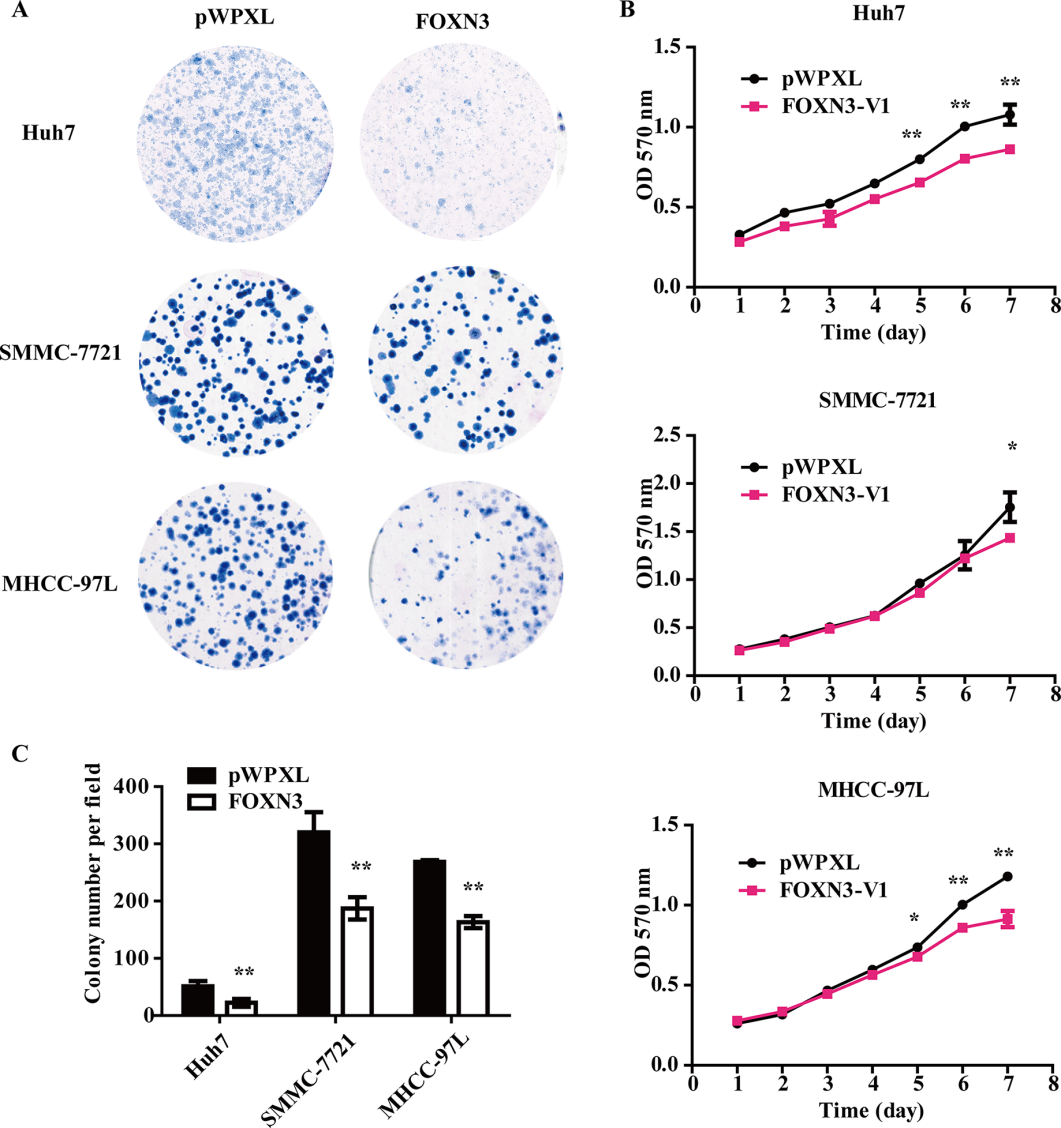
Overexpression of FOXN3 in HCC cells inhibits proliferation *in vitro* (**A**) The colony formation assay was performed in HCC cells stably transfected with FOXN3 or control plasmids. (**B**) Cell growth was analyzed at different time points (every one day interval) using an MTT assay. (**C**) Statistical analysis of the results shown in (A). *P < 0.05, **P < 0.01.

We measured the endogenous expression of the FOXN3 mRNA and protein in ten HCC cell lines (SMMC-7721, Huh7, PLC/PRF/5, LM3, 97L, SK-Hep1, HCC-LY5, HCC-LY10, 97H and Li7) and an immortalized liver cell line (L02) by western blotting and qRT-PCR (Figure 1E and 1F). FOXN3 expression was downregulated in SMMC-7721, Huh7 and MHCC-97L cells compared to other HCC cells. We then constructed a lentivirus vector containing the complete ORF of FOXN3 and established SMMC-7721-FOXN3, Huh7-FOXN3 and MHCC-97L-FOXN3 cell lines to further characterize the function of FOXN3 in HCC. We measured mRNA and protein levels using cells infected with empty vector as controls (Figure 1G and 1H). MTT and colony formation assays to investigate the effect of FOXN3 on proliferation indicated that FOXN3 inhibits HCC cell growth *in vitro* (Figure 2A, 2B and Supplementary Figure S1B).

### FOXN3 inhibits HCC tumorigenesis *in vivo*

**Figure 3:**
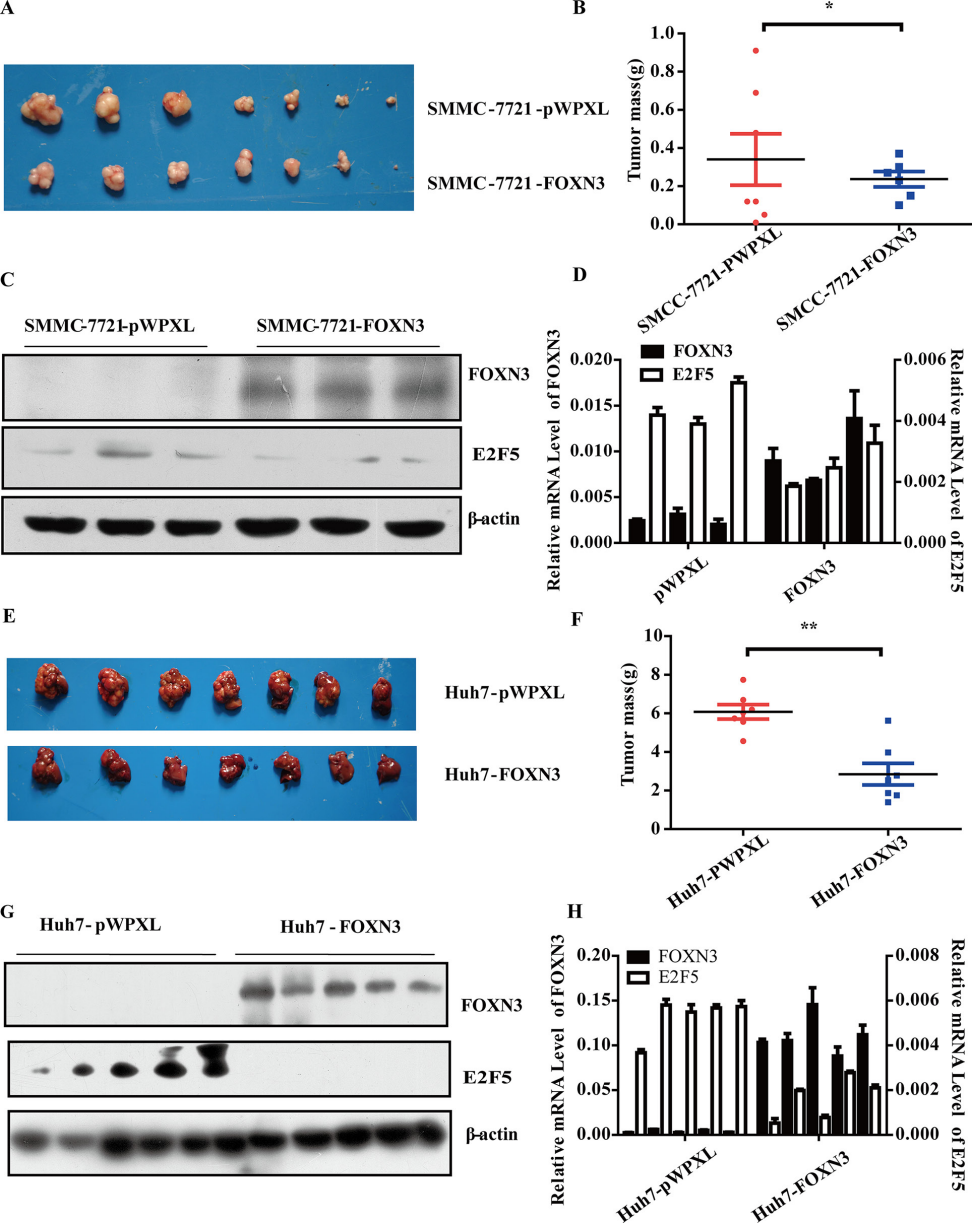
FOXN3 suppresses HCC tumorigenesis *in vivo* (**A**) SMMC-7721 cells stably transfected with FOXN3 were subcutaneously injected into nude mice; empty vectors were used as a control. After 7 weeks, the tumors were removed from the nude mice. (**B**) The weight of the liver tumors in (A). (**C**) Western blot analysis of FOXN3 expression in the samples in (A). (**D**) qPCR analysis of FOXN3 expression in the samples in (A). (**E**) MHCC-97L cells stably transfected with FOXN3 were orthotopically injected into the livers of nude mice; empty vectors were used as a control. After 4 weeks, the tumors were removed from the nude mice. (**F**) The weight of the liver tumors in (E). (**G**) Western blot analysis of FOXN3 expression in the samples in (E). (**H**) qPCR analysis of FOXN3 expression in the samples in (E). *P < 0.05, **P < 0.01.

We constructed two nude mouse models to further evaluate the potential role of FOXN3 in HCC tumorigenesis *in vivo* by subcutaneous injection of SMMC-7721-pWPXL/FOXN3 cells or liver orthotopic injection of Huh7-pWPXL/FOXN3 cells. When the mice became moribund, we removed the tumor. The weight of tumor revealed that FOXN3 inhibited HCC tumorigenesis significantly in both the subcutaneous model (*P* = 0.0132, Figure 3A and 3B) and the liver orthotopic model (*P* = 0.0004, Figure 3E and 3F) compared with the controls. These results are similar to the *in vitro* results and indicate an anti-proliferative role of FOXN3 in HCC.

### E2F5 is downregulated by FOXN3

**Figure 4:**
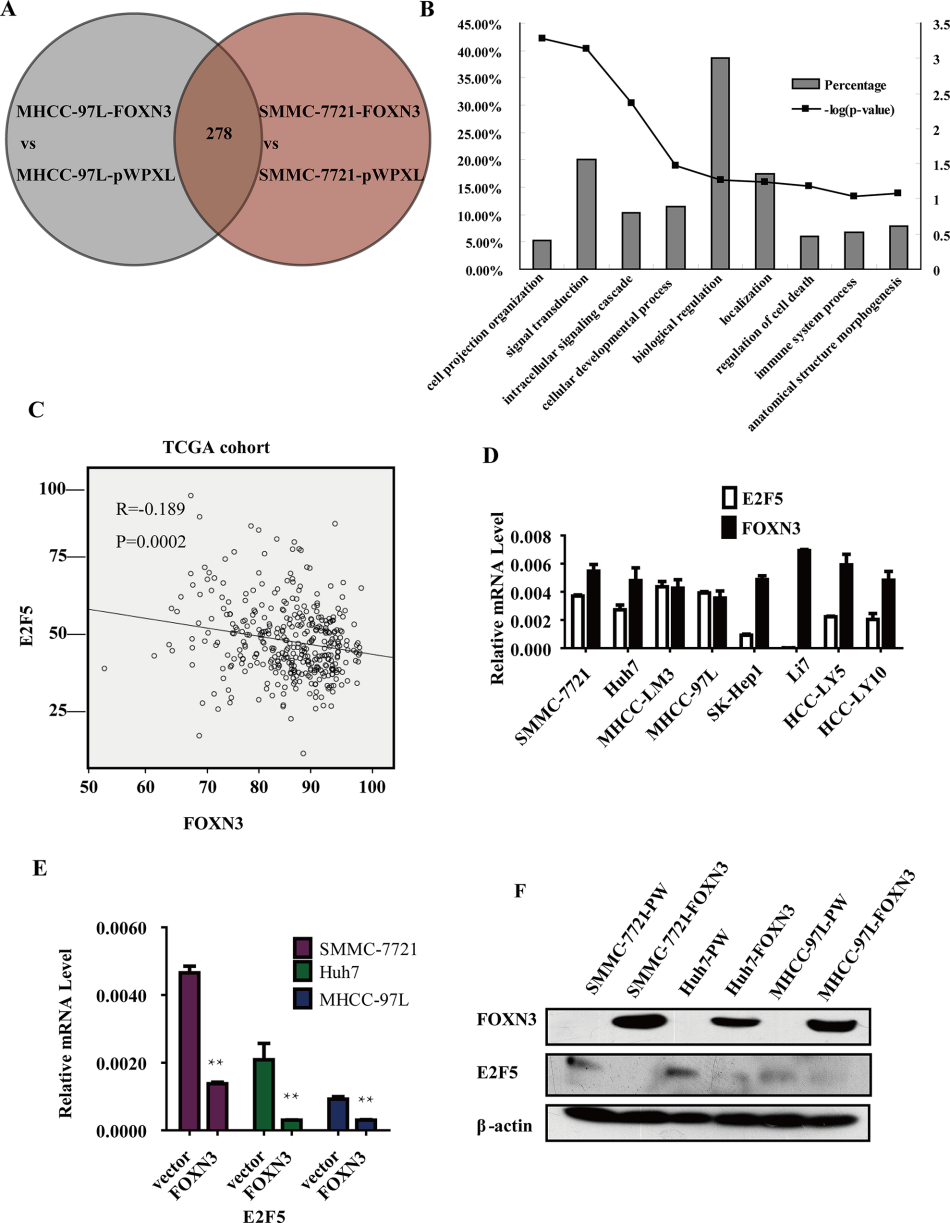
Microarray analysis reveals that FOXN3 acts as a transcriptional repressor of E2F5 (**A**) Venn diagram showing the overlap of the genes regulated by FOXN3 in the two cell lines. (**B**) The regulated genes were classified according to their functions using Functional Annotation Tool (DAVID Bioinformatics Resources 6.7, NIAID/NIH) (**C**) Negative correlation between FOXN3 and E2F5 mRNA levels in the TCGA cohort. (**D**) qPCR analysis of E2F5 and FOXN3 mRNA levels in the HCC cell lines. (**E**) and (**F**) Analysis of E2F5 mRNA and protein levels in HCC cells stably transfected with FOXN3. *P < 0.05, **P < 0.01.

To investigate the molecular mechanisms underlying the inhibitory effect of FOXN3 on HCC cell proliferation, cDNA microarrays were performed to identify differentially expressed genes in FOXN3-overexpressing SMMC-7721 and MHCC-97L cells compared with the control. A total of 278 genes were differently expressed in both HCC cell lines with a fold change greater than 2 (Figure 4A). Next, we classified the 278 genes by function using the Functional Annotation Tool (DAVID Bioinformatics Resources 6.7, NIAID/NIH), which indicated roles of these genes in signal transduction, biological regulation, location and cellular developmental processes (Figure 4B). Based on their functions, we chose the most obvious differentially expressed genes (IFT88, ANXA9, E2F5, RASSF4 and COL8A1). Then, we confirmed the regulation of these genes in three FOXN3-overexpressing HCC cell lines (Figure 4E and Supplementary Figure S2A). We detected the correlation between these genes and FOXN3 in 372 HCC tissues by analyzing the TCGA data. Only E2F5, a probable oncogene in HCC, was consistently downregulated in the FOXN3-overexpressing HCC cell lines and significantly negatively correlated with FOXN3 in the 372 HCC tissues (*P* = 0.0002, *R* = −0.189, Figure 4C and Supplementary Figure S2B). E2F5 mRNA expression was also negatively correlated with FOXN3 mRNA expression in these HCC cell lines and HCC tissues (Figure 4D and Supplementary Figure S3A). Based on the transcriptional repressor role of FOXN3 and the results of the expression analysis of E2F5 and FOXN3, we hypothesized that E2F5 is the target of FOXN3. We therefore measured the expression of the E2F5 protein in the FOXN3-overexpressing HCC cell lines (Figure 4F). We also measured the expression of E2F5 mRNA in 60 pairs of HCC tissues and matched non-tumorous liver tissues by qRT-PCR. In contrast to FOXN3, E2F5 was upregulated in primary HCC tissues compared with non-tumorous liver tissues (*P* < 0.0001, Supplementary Figure S3B and Supplementary S3C). These results demonstrate that FOXN3 downregulates the expression of E2F5 at both the mRNA and protein levels.

### FOXN3 directly binds to the E2F5 promoter and suppresses its activity

**Figure 5:**
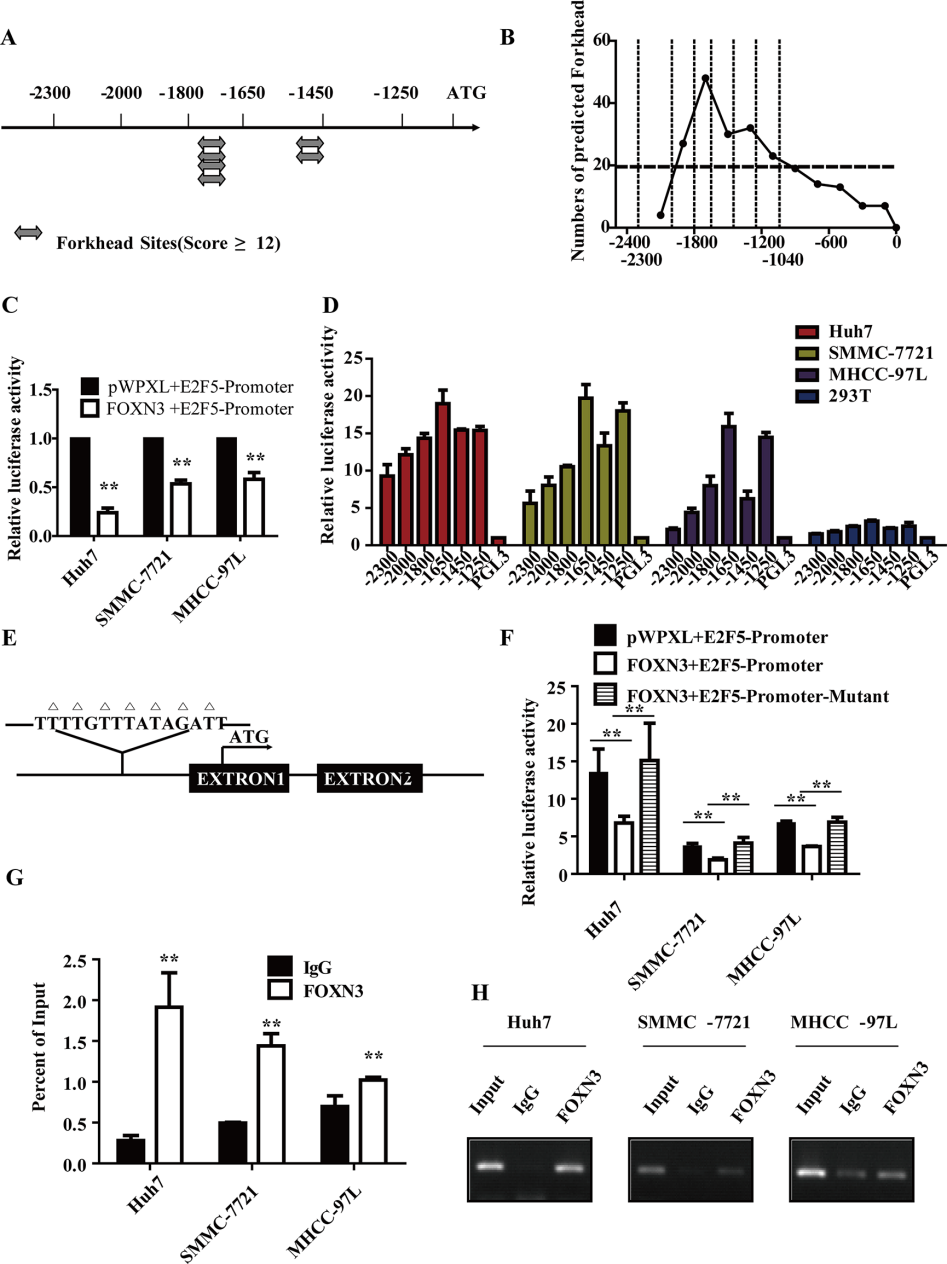
FOXN3 directly binds to and represses the E2F5 promoter in HCC cells (**A**) Identification of potential forkhead binding sites in the E2F5 promoter with a score greater than 12 according to the JASPAR database. The analysis was performed by selecting all FOX family transcription factors from the JASPAR database as the template. (**B**) The numbers of all potential forkhead binding sites in the different regions of the E2F5 promoter. (**C**) Activity of the E2F5 promoter after transfection of FOXN3 into HCC cells. (**D**) Activity of the E2F5 promoter deletion mutants in Huh7, SMMC-7721, MHCC-97L and 293T cells. (**E**) The predicted FOXN3 binding site on the E2F5 promoter; the mutant is marked. (**F**) The activities of the E2F5 promoter and the mutant promoter after transfection of HCC cells with FOXN3. (**G**) The binding of FOXN3 to the E2F5 promoter in HCC cells was analyzed by chromatin immunoprecipitation using antibodies to FOXN3. A negative control with an irrelevant antibody (IgG) was included for comparison. qPCR was used to analyze the E2F5 promoter. (**H**) Agarose gel electrophoresis was used to analyze the crosslinking status. *P < 0.05, **P < 0.01.

We analyzed all potential forkhead binding sites along the E2F5 promoter from −2.3 kb to 0 kb of the transcription start site using the JASPAR database and the FOX family transcription factor matrix (Figure 5A and 5B). To determine whether FOXN3 downregulates E2F5 via direct binding to the promoter of E2F5, we constructed a plasmid carrying the E2F5 promoter region from −2300 bp to −1040 bp, which contains the main binding sites. Reporter assays revealed that the relative luciferase activity of the promoter was inhibited when FOXN3 was overexpressed in HCC cells (Figure 5C). This result suggests that the promoter activity of E2F5 was directly or indirectly downregulated by FOXN3. Then, we successively deleted sequences from the E2F5 promoter and investigated the activity of these promoters. The activity of the promoter was increased when the −1800 bp/−1650 bp sequences were deleted (Figure 5D). Based on this result, we suspected that this region might contain the binding site for the transcriptional repressor FOXN3. After analyzing the possible FOXN3 binding sites using the FOX family transcription factor, we constructed a plasmid containing a mutant of the potential binding site at −1800 bp (Figure 5E). The reporter assay and chromatin immunoprecipitation assay verified that this site was the FOXN3 binding site on the E2F5 promoter (Figure 5F, 5G and 5H). Together, these results suggest that FOXN3 inhibits the expression of E2F5 by directly binding to its promoter in HCC cells.

### Reintroduction of E2F5 impairs the FOXN3-induced suppression of HCC cell proliferation

**Figure 6:**
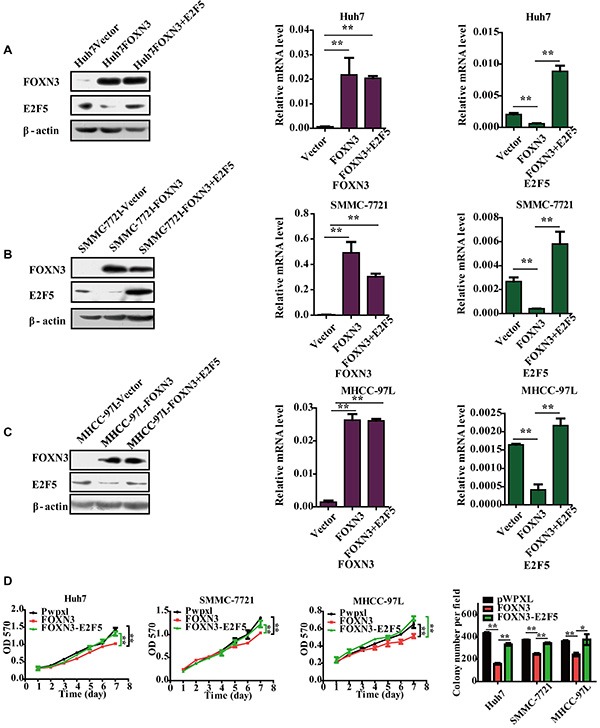
Reintroduction of E2F5 impairs the FOXN3-induced suppression of HCC cell proliferation (**A**), (**B**) and (**C**) Western blot and qPCR analyses of FOXN3 or E2F5 expression in E2F5-trasfected HCC cells or controls. (**D**) Cell growth at different time points (every one day interval) was analyzed using an MTT assay and colony formation assay. *P < 0.05, **P < 0.01.

Because transcription factors exert their functions by regulating the expression of their target genes, we evaluated the role of E2F5 in the FOXN3-induced suppression of HCC cell proliferation because E2F5 is the target of FOXN3 in HCC cells. We restored the expression of E2F5 in FOXN3-overexpressing HCC cells by transfecting an E2F5 expression plasmid in FOXN3-overexpressing HCC cell lines (Figure 6A, 6B and 6C). In this functional rescue experiment, reintroduction of E2F5 obviously reversed the FOXN3-induced repression of cell proliferation in Huh7, SMMC-7721 and MHCC-97L cells (Figure 6D). These data clearly demonstrate that FOXN3 inhibits the proliferation of HCC cells by decreasing E2F5.

## DISCUSSION

HCC is one of the deadliest types of human cancer. Despite advances in the diagnosis and treatment of HCC, the understanding of the underlying molecular mechanisms of HCC remains lacking. Transcription factors play vital roles in cancer progression, and studies of transcription factors may provide insights into the molecular mechanisms of HCC and reveal new therapeutic targets for the effective treatment of HCC patients.

As a subclass of FOX family transcription factors, FOXN has six members: FOXN1, FOXN2 (HTLF), FOXN3 (CHES1), FOXN4, FOXN5 (FOXR1) and FOXN6 (FOXR2). In FOXN1 knockout mice, an inability to attract lymphoid precursors to the thymus primordium and aberrant epithelial morphogenesis are observed. Furthermore, FOXN1 is regulated by Wnt glycoprotein [12]. FOXN2, also known as human T-cell leukemia factor (HTLF), was identified as a cellular gene that may function as a transcriptional regulator of the human T-cell leukemia virus long terminal repeat (HTLV-I LTR) [13]. FOXN4 controls the formation of amacrine and horizontal cells by upregulating the expression of retinogenic factors [14]. FOXN5/R1 was first reported as a candidate tumor suppressor gene [15]. FOXN6/R2 promotes cell proliferation and malignancy in HCC. Wang suggested that FOXN6/R2 is a novel promising therapeutic target for HCC [16].

As a member of the FOXN subclass, FOXN3 plays an indispensable role in cellular developmental processes. FOXN3 restores S-phase arrest in MnnI mutant Drosophila by functioning as a DNA-damage response protein [17]. FOXN3 is a direct target of and is downregulated by miR-574-5p to promote the progression of human lung cancer [18]. Moreover, FOXN3 inhibits cell proliferation by repressing PIM2 and protein biosynthesis [19]. FOXN3 also suppresses the expression of N-cadherin mRNA and protein by transcriptionally repressing N-cadherin promoter activity [20]. However, the role of FOXN3 in HCC tumorigenesis remains poorly understood. The current study is the first to confirm an important role of FOXN3 in HCC. Our study demonstrates that FOXN3 functions as a transcriptional repressor and inhibits E2F5 expression in HCC.

Because FOXN3 has two variants, we constructed two different FOXN3 plasmids. FOXN3 variant2 was expressed at very low levels in the HCC cell lines (Figure 1F). Therefore, we focused on FOXN3 variant1 (FV1), of which open reading frame (ORF) is longer and contains variant2 (FV2).

E2F5, an important member of the E2F family, is a potential oncogene in breast cancer [21], ovarian cancer [22], HCC [23], esophageal squamous cell carcinoma [24], prostate cancer [25], and colorectal cancer [26]. In the present study, we also observed that the expression of E2F5 is upregulated in HCC tissues compared with matched non-tumorous liver tissues.

Although exact binding sites for FOXN3 have not been clearly established, we predicted potential binding sites by analyzing forkhead binding sites using the FOX family transcription factors matrix. In this study, we observed that FOXN3 inhibited the proliferation of HCC cells *in vitro* and *in vivo* by suppressing E2F5 expression via direct binding to the promoter of E2F5. These results provide potential targets for the prevention and treatment of HCC.

## MATERIALS AND METHODS

### Patient material

Sixty pairs of cancer tissues and matched adjacent liver tissue samples of patients with HCC were obtained from the Qidong Liver Cancer Center. The University Ethical Committee approved the collection of fresh tumor tissue samples for clinical analysis. The tissue sections and specimens were prepared by pathologists, snap frozen and subsequently stored at −80°C.

### Cell culture

The cell lines used in this study, SMMC-7721, Huh7 and MHCC-97L, were obtained from the Cell Bank of the Institute of Biochemistry and Cell Biology (Shanghai, China) or the Liver Cancer Institute of Zhongshan Hospital at Fudan University (Shanghai, China), and routinely cultured at 37°C in a humidified atmosphere with 5% CO2. All cell lines were incubated in Dulbecco's Modified Eagle's Medium (Sigma-Aldrich, St. Louis, MO) supplemented with 10% fetal bovine serum (FBS) (HyClone, Logan, UT) and 1% penicillin/streptomycin (Sigma, USA).

### Plasmids

The sequences of the full-length human FOXN3 gene were amplified by PCR and cloned into pWPXL at the MluI and EcoRI sites. The sequences of the full-length human E2F5 gene were amplified by PCR and cloned into pWPXL at the BamHI and EcoRI sites. The sequence of the full-length E2F5 promoter was amplified from −2300 bp to −1040 bp; the deletion plasmids were amplified from −2000 bp, −1800 bp, −1650 bp, −1450 bp and −1250 bp to −1040 bp. The mutant was generated by mutating the DNA binding site (−1684 bp to −1670 bp). These promoter sequences were all cloned into the pGL3-enhancer at the BglII and MluI sites.

### Primers

The RT and clone primers used in this study were designed using NCBI/Primer-BLAST (http://www.ncbi.nlm.nih.gov/tools/primer-blast/index.cgi?LINK_LOC=BlastHome). The primers are listed in Supplementary Table S1 and Supplementary Table S2.

### Expression data

A total of 373 liver cancer patients in TCGA (https://tcga-data.nci.nih.gov/tcga/tcgaHome2.jsp, version on 2015-02-24) database (hereafter referred to as the TCGA cohort) were enrolled in this study and analyzed. Patients who lived longer than 7 years or less than 5 months were excluded from the survival analyses, with a FOXN3 expression cut point of 84. All 373 patients from TCGA who had E2F5 and FOXN3 mRNA expression data were used to analyze the correlation between E2F5 and FOXN3. A total of 50 patients from TCGA with FOXN3 mRNA expression data in both their cancerous tissues and matched non-tumorous liver tissues were used to analyze FOXN3 mRNA levels.

### Chromatin immunoprecipitation, lentivirus production, and cell transfection

Chromatin immunoprecipitation (Chip), lentivirus production, and cell transfection were performed as previously described [27–29].

### Western blotting

Proteins were extracted from tissue lysates or cell lines, separated by SDS-PAGE, and transferred to nitrocellulose or polyvinylidene difluoride membranes. The anti-FOXN3 polyclonal antibody (Ab50756) and the anti-E2F5 polyclonal antibody (Ab176017) were purchased from Abcam (Cambridge, UK). The anti-β-actin antibody (Cat No. A3854) was purchased from Sigma-Aldrich (Missouri, USA).

### Luciferase assay

Cell lines were maintained in DMEM and cotransfected with the corresponding reporter plasmids after reaching approximately 90% confluence. Cells were also cotransfected with a PRL-TK reporter construct. After 48 hours, luciferase activity was measured using the Dual-luciferase reporter assay kit (Promega, USA) according to the manufacturer's instructions.

### Cell proliferation assay

A total of 2,000 cells were plated on a 96-well plate. After incubation for 24 hours, MTT reagent (5 mg/ml, Sigma-Aldrich, USA) was added to the medium in each well and incubated for 4 hours at 37°C. Then, optical density (OD) values were measured at 570 nm for the cell proliferation assay.

### Tumor xenograft models

Two different types of mouse models were used to observe the effects of FOXN3 on HCC cells. For the subcutaneous model, groups of 6- to 8-week-old BALB/c (nu/nu) mice were injected with suspended HCC cells (2×10^6^ SMMC-7721 cells). Approximately 7 weeks after HCC cell inoculation, the mice were euthanized when they became moribund. For the liver orthotopic model, suspended HCC cells (2 × 10^6^ Huh7 cells) were injected into the liver, and the mice became moribund after 4 weeks.

### Statistical analysis

All data are presented as the mean ± standard deviation (SD) from at least three independently. *P* < 0.05 was considered statistically significant. Statistical analyses (comparisons between two groups) were performed using Student's *t* test. Kaplan-Meier analysis was used to the correlation compare between two groups.

## SUPPLEMENTARY MATERIALS FIGURES AND TABEL


