# Supplementary material for: The transcription factor FOXN3 inhibits cell proliferation by downregulating E2F5 expression in hepatocellular carcinoma cells
Source: Oncotarget. 2016 Jun 2;7(28):43534–45. doi: 10.18632/oncotarget.9780 (PMC5190042; doi:10.18632/oncotarget.9780)
Supplement: Supplementary file 1 [file oncotarget-07-43534-s001.pdf]

The transcription factor FOXN3 inhibits cell proliferation by downregulating E2F5 expression in hepatocellular carcinoma cells

Supplementary Materials

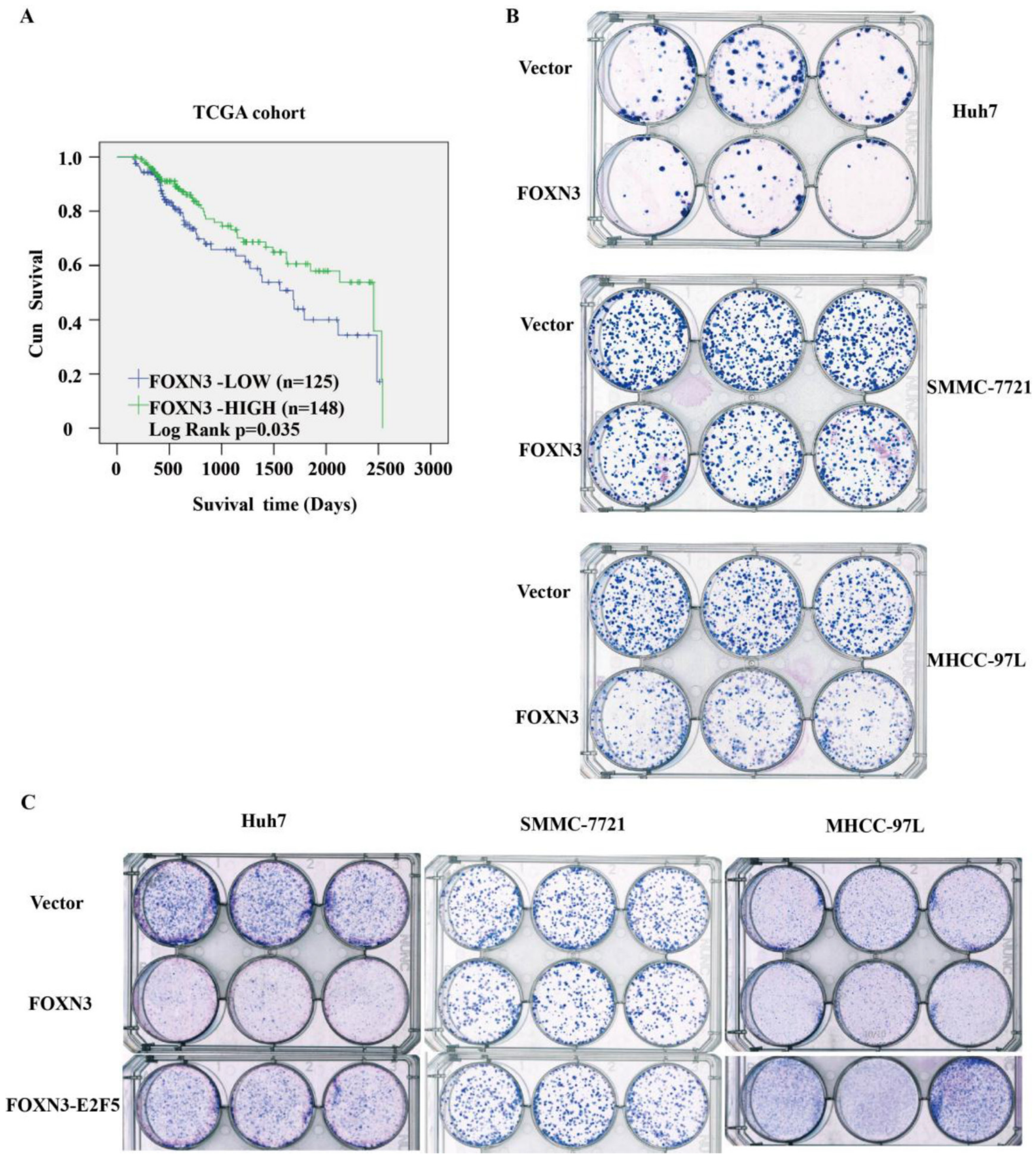

Supplementary Figure S1: FOXN3 plays an antiproliferative role in HCC. (**A**) Kaplan-Meier analysis of the correlation between FOXN3 expression and overall survival in TCGA cohort. Log-rank test was used to determine statistical. (**B**) Colony formation was performed in HCC cells stably transfected with FOXN3 or the control. (**C**) Colony formation was performed in HCC cells reintroducted of E2F5 or the control.

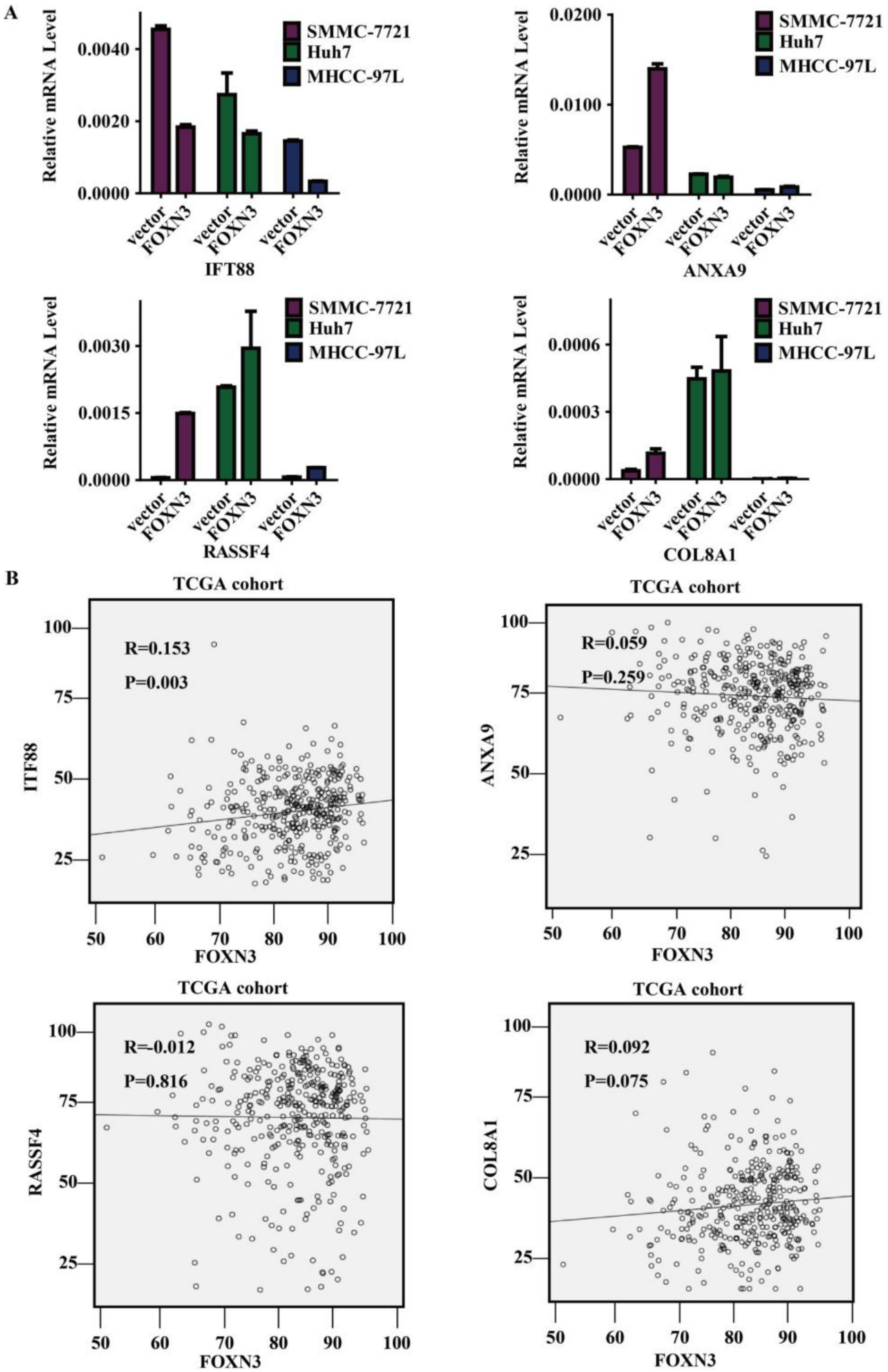

Supplementary Figure S2: We detected other gene chosen from Microarray analysis as the target of FOXN3 except E2F5. (**A**) qRT-PCR analysis of the mRNA levels for the indicated gene in HCC cell lines overexpressing FOXN3. (**B**) Analysis the correlation between FOXN3 and the indicated genes in 373 HCC tissues using data from TCGA.

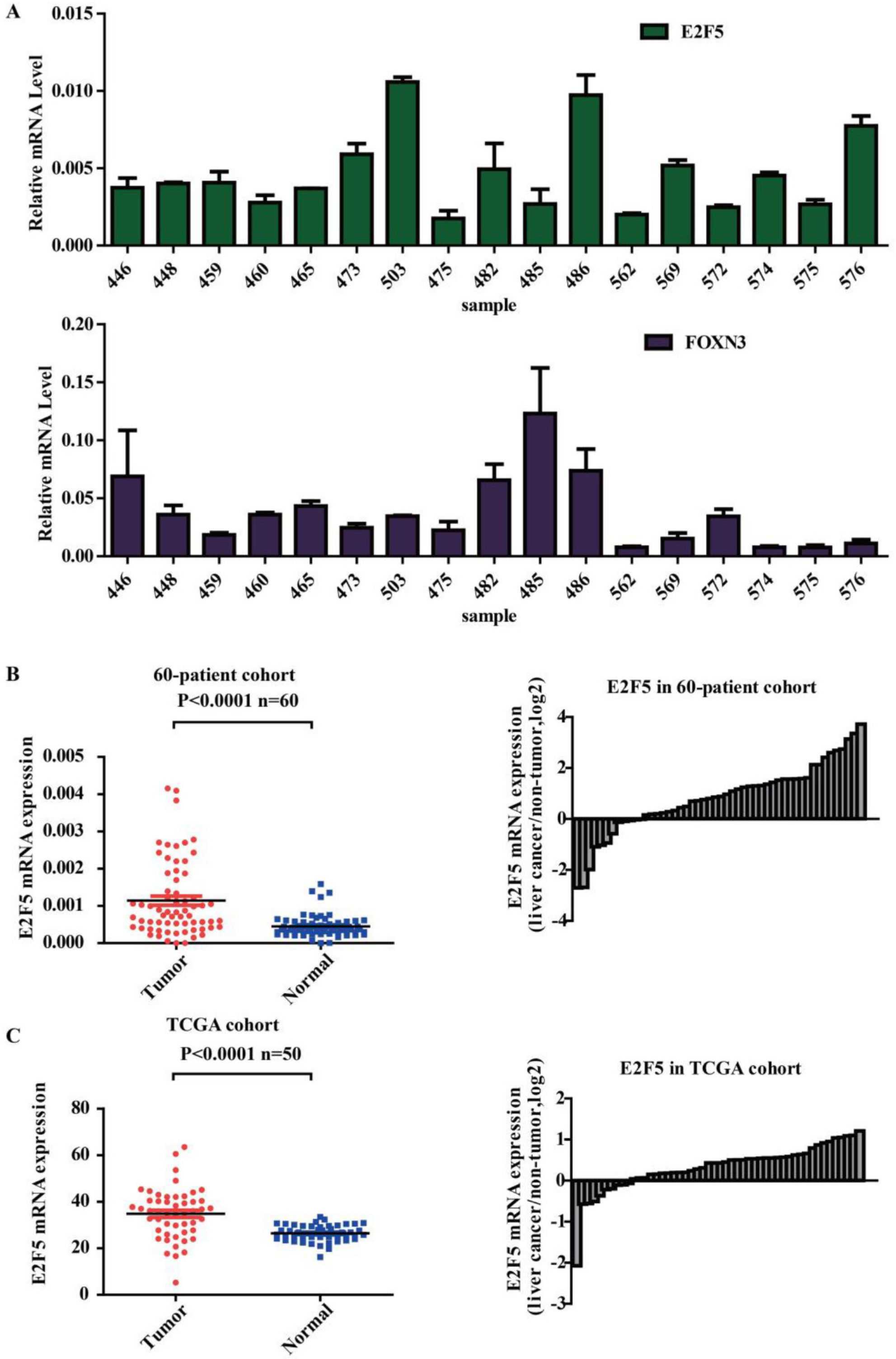

Supplementary Figure S3: The expression of E2F5 was upregulated in HCC tissues compared with the matched non-tumorous liver tissues and partly negative correlated with FOXN3 in HCC tissues. (**A**) qRT-PCR analysis of the mRNA levels for E2F5 and FOXN3 in several HCC tissues. (**B**) qRT-PCR analysis of the mRNA expression of E2F5 in 60-patient cohort. (**C**) Expression levels of E2F5 in TCGA cohort.

Supplementary Table S1: Primer for qRT-PCR and CHIP

Name	Primer Sequence
FOXN3-F	5′-TCTGACATGCCCTACGATGC-3′
FOXN3 -R	5′-CTATGCACCACAACGACCCT-3′
E2F5-F	5′-TCTTCAGCAGGATCTATTAGTGG-3′
E2F5-R	5′-TGTAGTCATCTGCCGGGGTA -3′
IFT88-F	5′-GTGCGTGGAAGTGGTGAAAG-3′
IFT88 –R	5′-AGGGCTGAGAGATTGGTTGC-3′
ANXA9-F	5′-CTCGGCCTAGCTTCGGTGAT-3
ANXA9-R	5′-GACTGGCAATCCCCTTTCAC-3′
RASSF4-F	5′-AGTCCATTCAGAAGTCGGAGC-3
RASSF4-R	5′-ATGGCTCCTTTAGAGGGCAG-3
COL8A1-F	5′-TATAAATGTGATGGCTGTGCTGC-3
COL8A1-R	5′-CAGCGGCTTGATCCCATAGT-3
CHIP-F	5′- GCCTTTCTTGGATAGGTTGTT -3
CHIP-R	5′- ACTAGTAAAGCTGAACACACCA -3

F, forward primer; R, reverse primer; RT, reverse-transcription primer.

Supplementary Table S2: Primer for clone

Name	Primer Sequence
FOXN3-F	5′- TACACGCGTATGGGTCCAGTCATGCCTCC -3′
FOXN3 -R	5′- CGCGCGAATTCTTAATTTTTTGTGGTTTCCT -3′
E2F5-F	5′- TATATTAGGATCCATGGCGGCGGCAGAGCCCGCGA -3′
E2F5-R	5′- GGCCGGAATTCCTAATAATTTAGTATCTGG -3′
E2F5-Pro2300-F	5′- CGACGCGTCGATCGTGAAGATTGAGCCCTC -3′
E2F5-Pro2000-F	5′- CGACGCGTGTGTTATTAATTCCTGTC -3′
E2F5-Pro1800-F	5′- CGACGCGTATGGTTGTATCAACTGAA -3
E2F5-Pro1650-F	5′- CGACGCGTCAGCTTTACTAGTTAATG -3′
E2F5-Pro1450-F	5′- CGACGCGTTGCAGTGGTGGCATCTCG -3
E2F5-Pro1250-F	5′- CGACGCGTCTGCCTCGGCCTCCCAAA -3
E2F5-Pro-mutant-F	5′- CGACGCGTATGGTTGTATCAACTGAATTTTTCATGTTTTACTTCAGATTTT CAATTTTAAGTCTCGCTCACAAACTTGATTAAAATAAAGGTTA -3
E2F5-Pro-R	5′- GGAAGATCTAGATCTGAGTAAAAGAAAG -3

F, forward primer; R, reverse primer; RT, reverse-transcription primer
